# Modulation of Activity in Human Visual Area V1 during Memory Masking

**DOI:** 10.1371/journal.pone.0018651

**Published:** 2011-04-15

**Authors:** Markus H. Sneve, Dag Alnæs, Tor Endestad, Mark W. Greenlee, Svein Magnussen

**Affiliations:** 1 Center for the Study of Human Cognition, Department of Psychology, University of Oslo, Oslo, Norway; 2 Department of Experimental Psychology, University of Regensburg, Regensburg, Germany; Tel Aviv University, Israel

## Abstract

Neurons in the primary visual cortex, V1, are specialized for the processing of elemental features of the visual stimulus, such as orientation and spatial frequency. Recent fMRI evidence suggest that V1 neurons are also recruited in visual perceptual memory; a number of studies using multi-voxel pattern analysis have successfully decoded stimulus-specific information from V1 activity patterns during the delay phase in memory tasks. However, consistent fMRI signal modulations reflecting the memory process have not yet been demonstrated. Here, we report evidence, from three subjects, that the low V1 BOLD activity during retention of low-level visual features is caused by competing interactions between neural populations coding for different values along the spectrum of the dimension remembered. We applied a memory masking paradigm in which the memory representation of a masker stimulus interferes with a delayed spatial frequency discrimination task when its frequency differs from the discriminanda with ±1 octave and found that impaired behavioral performance due to masking is reflected in weaker V1 BOLD signals. This cross-channel inhibition in V1 only occurs with retinotopic overlap between the masker and the sample stimulus of the discrimination task. The results suggest that memory for spatial frequency is a local process in the retinotopically organized visual cortex.

## Introduction

The primary visual cortex (V1) is the first cortical area where neurons show selective processing of elemental visual features such as orientation, color, and spatial frequency [1], [2], [3]. Psychophysical studies have shown that detailed representations of such basic stimulus features can be retained in memory for seconds and even minutes with little loss of information [4], [5]. This ‘sensory working memory’ [6] has recently been investigated in a series of functional magnetic resonance imaging (fMRI) studies, probing delay period activity in memory tasks for orientation [7] and color [8] using multi-voxel pattern analysis (MVPA [9]). In accordance with earlier findings on nonhuman primates [10], [11], and current neural models of visual working memory [12], [13], the researchers managed to decode stimulus-specific properties from V1 during retention, suggesting that memory for low-level visual features recruits the same neural populations that were involved in their perceptual encoding.

Interestingly, none of these fMRI studies find consistent amplitude changes in the blood-oxygenation level-dependent (BOLD) response in V1 during memory maintenance (i.e. activity falls to baseline levels; see also [14]). This lack of correspondence between the MVPA findings and the results from the univariate analyses may result from suppression of neurons tuned to non-remembered values of the task relevant feature – in effect canceling out memory-related increases in neural activity on the population (i.e. voxel) level. Here we investigate and take advantage of this lateral suppression effect to further explore the involvement of V1 and other early visual areas in visual sensory working memory, using fMRI and univariate analyses.

When a task-irrelevant stimulus is presented at some point during a delayed discrimination task but outside the temporal reach of conventional sensory masking [15], memory performance may suffer. This ‘memory masking’ effect occurs when the mask differs from the memory item along the dimension to be remembered: When instructed to remember the spatial frequency of a grating, no interference is observed when the spatial frequency of the mask matches the frequency to be remembered, but with a masker twice or half the frequency of the memory item (±1 octave), memory is substantially impaired. No masking is observed when the two stimuli differ along a task-irrelevant feature [16], [17], [18], [19]. The observation of interference within, but not between dimensions, has led to the formulation of a model consisting of narrowly tuned, feature-specific filters arranged in laterally inhibitory networks and located in early visual areas [4], [20].

While the above-mentioned fMRI studies [7], [14], [21] use simple delayed discrimination tasks with long delay intervals between the two stimuli to be discriminated, we take advantage of the memory masking paradigm in which the strength of a memory representation is modulated from trial to trial. Based on our model, we predict that the introduction of a memory masker will produce a weaker BOLD response in visual areas involved in the online storage of spatial frequency information.

In an additional experiment, we test the spatial specificity of this memory modulation. A recent fMRI study [21], also using a MVPA approach to investigate visual sensory working memory maintenance, finds feature-specific activity patterns in ipsilateral V1 (relative to stimulus position), which would seem to imply that memory representations are not confined retinotopically. Other behavioral studies, both in nonhuman primates [22] and in humans [23], have, however, found reductions in delayed discrimination performance with stimuli presented to different locations – the critical spatial separation corresponding to the receptive field size of neurons involved in their encoding. By presenting the memory masker to a different position in the visual field than the stimuli to be discriminated, we examine the spatial extent of the suggested suppressive mechanisms.

## Materials and Methods

### Ethics Statement

The study has been approved by the regional ethics committee (Regional Committee for Medical and Health Research Ethics, South-East Norway). All participants gave their written informed consent, signing a statement approved by the Regional Committee for Medical and Health Research Ethics (South-East Norway), prior to commencing the study.

### Participants

Three experienced psychophysical observers took part in the experiments (all males, 27–30 years of age). They were thoroughly trained on the experimental tasks before the reported data were collected. The observers first participated in two psychophysical experiments in which behavioral measurements, as well as an initial session of estimating task discrimination thresholds, were conducted in a psychophysics laboratory. The fMRI part of the study was comprised of two experiments, in addition to a localizer session to map individual regions of interest (ROI), and a retinotopic mapping session to define visual areas.

### Stimuli and stimulus presentation

In the psychophysical experiments, the stimuli were presented on a calibrated 19-inch Eizo FlexScan L768 monitor (Eizo Nanao Corporation, Ishikawa, Japan). In the fMRI sessions, the stimuli were back-projected on a screen inside the scanner by use of a modified F20 sx+ DLP® digital projector (Projectiondesign, Fredrikstad, Norway). Screen resolutions were set at 1400×1050 pixels.

#### Main experiments

In all experiments, the stimuli were Gabor gratings with a 2D-patch of sinusoidal grating that subtended 10° of visual angle. The phase of the sinusoid varied randomly between trials. The sinusoid had a maximum Michelson's contrast of 0.9, and was tapered with a Gaussian kernel with a standard deviation of 1.25°. Gabor stimuli were presented at four different positions in the experiments, located in each of the four visual field quadrants. The distance between fixation and the center of the Gabors was 6° of visual angle for all four positions.

#### fMRI localizer session

For the fMRI localizer session, stimuli were radial black and white checkerboards at maximum contrast, centered at the four positions of interest. The checkerboards had diameters of 5.2° visual angle (corresponding to the area of the Gabor gratings with a Michelson's contrast over 0.1) and were scaled relative to fixation following the linear cortical magnification factor [24], with a fixation cross indicating the center of the display in all sessions.

#### Retinotopic mapping session

Standard checkerboard stimuli (rotating wedges, expanding ring [25]) were used in the retinotopic mapping session.

### Procedure, psychophysical experiments

#### Threshold estimation

Before being tested in each of the two experiments, the participants went through 6 runs × 40 trials of an adaptive maximum likelihood procedure, QUEST, as implemented in the Psychophysics Toolbox 3 extensions [26], [27] for MatLab (MathWorks, Natick, MA) in order to estimate spatial frequency discrimination thresholds. A two-interval forced-choice delayed discrimination task with a delay between the two stimulus intervals of 3 seconds (3 runs) or 9 seconds (3 runs) was used. The desired threshold estimate was set to a hit rate (percentage correct) of 75% or 85%, respectively, and the spatial frequency differences that, for each participant, produced these hit rates were used as individual difference levels in the two tasks constituting a trial in the experiments (see below).

#### Main experiments

**Figure 1:**
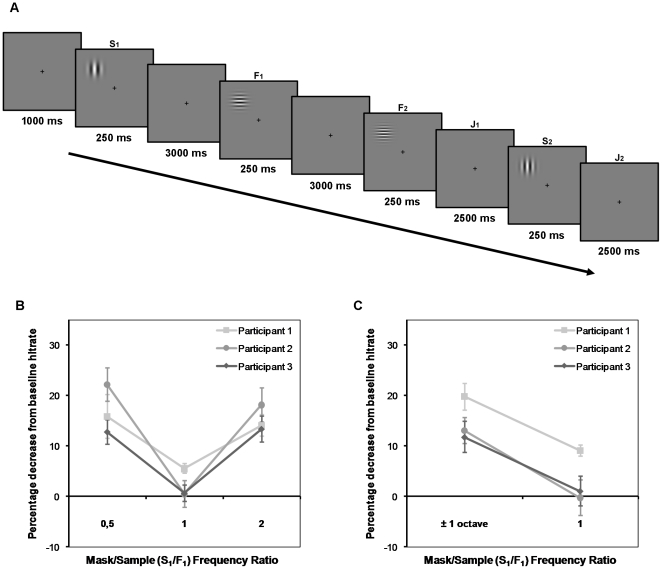
Experiment 1. A) All stimuli in a trial were presented to the same quadrant of the visual field. Participants had to remember stimulus S_1_ (the mask) and, after a short delay, F_1_ (the sample) to perform two delayed discriminations: J_1_ (comparing F_1_ and F_2_), and J_2_ (comparing S_1_ and S_2_). The F_1_–F_2_ comparison was the main task of interest and the spatial frequencies of the stimuli differed at the individually estimated 75% hit rate level. The S_1_–S_2_ comparison was introduced to ensure that subjects actively tried to remember the mask and differed at the estimated 85% hit rate level. The mask and sample always had orthogonal orientations to avoid priming effects. B) Behavioral results from psychophysical sessions. The graphs are normalized changes in F_1_–F_2_ discrimination for the three participants with respect to their individual baseline measure. C) Behavioral results from fMRI Experiment 1. Note that the two Mask/Sample (S_1_/F_1_) Frequency Ratios differing from one in the psychophysical experiment were combined in the analysis of the fMRI experiment. Error bars represent ±1 SE.

**Figure 2:**
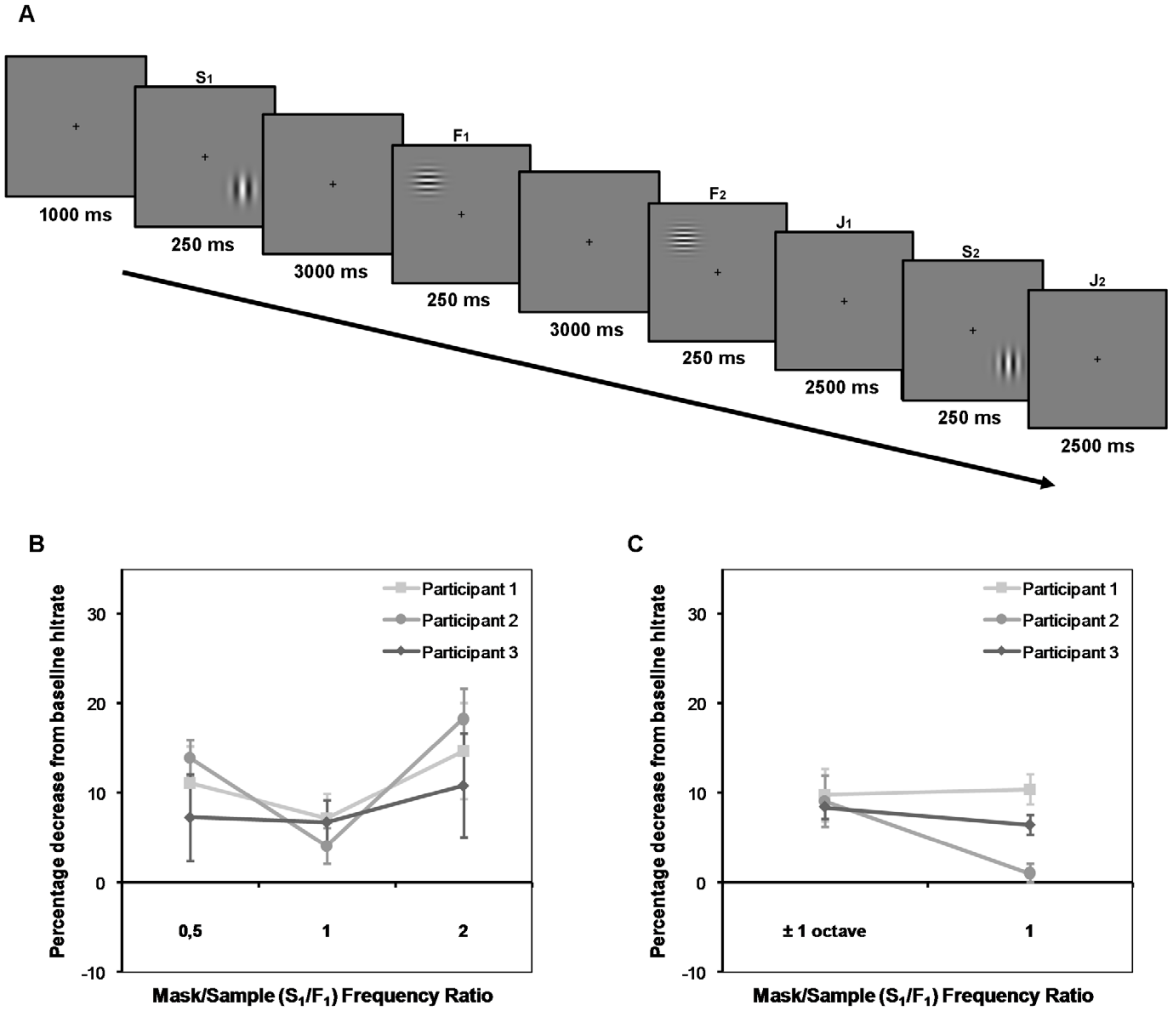
Experiment 2 – see Fig. 1A for more details. A) Experiment 2 was identical to Experiment 1 with the exception that Stimulus F_1_ and F_2_ were always presented at the far opposite position of stimulus S_1_ and S_2_. B) Behavioral results from psychophysical sessions. C) Behavioral results from fMRI Experiment 2.

The two experiments were modified versions of the memory masking paradigm used by Lalonde and Chaudhuri [16]. This version of the paradigm differs from the original (e.g. [18]) in that the interfering stimulus (the mask) is presented before the sample stimulus to be remembered (see Fig.1A). Additionally, the mask is involved in a second, much easier, discrimination task to ensure that it is actively encoded. To avoid confusion of the stimuli in a trial and to control for the possibility of priming effects, the orientations of the gratings in the S1–S2 task (first and fourth stimulus in a trial) and the F1–F2 task (second and third stimulus in a trial) were always orthogonal to each other. The specific orientations (vertical (90°) or horizontal (0°)) of the two stimulus pairs were randomized across trials. The individually estimated spatial frequency difference thresholds (difference producing 75% hit rate at an ISI of 3 seconds, corresponding to the F1–F2 interval; difference producing 85% hit rate at an ISI of 9 seconds, corresponding to the S1–S2 interval) were used as the percentage difference between the stimuli in the two tasks. The test stimuli (F2 and S2) could increase or decrease with this percentage, and the participants knew that both directions of change occurred with equal probability. The S1–S2 discrimination was thus a markedly easier task than the F1–F2 discrimination, and the S2 stimulus was mainly included to ensure active encoding of the mask stimulus (S1). The spatial frequency relationship between the mask (S1) and the sample stimulus (F1) varied from trial to trial in three established ratios: the spatial frequency of S1 could be the same as for F1 (mask/sample ratio (MSR) = 1), one octave above F1 (MSR = 2), or one octave below F1 (MSR = 0.5). The stimuli used in the experiments varied across a spatial frequency range of 1.2–6 cycles per visual degree (c/deg), with an average frequency of 3 c/deg. All spatial frequencies were counterbalanced across stimulus pairs. One session of the experiment consisted of 216 trials, which were divided into 3 runs separated by breaks. All participants were tested over 3 sessions on each of the two experiments, which produced 216 observations per MSR per experiment. The two experiments only varied in the relative positions of the S1–S2 task and the F1–F2 task within a given trial. In Experiment 1, all stimuli in a trial were presented in the same stimulus position (Fig.1A). In Experiment 2, stimulus S1 and S2 were presented in one quadrant, while F1 and F2 were presented in the far opposite quadrant (Fig. 2A). All conditions were sampled equally often and in a randomized order within an experimental run.

### Procedure, fMRI experiments

#### fMRI localizer session

All observers participated in a localizer session to identify individual ROIs in early visual areas. Flickering checkerboard patches were presented with a flickering rate of 10 Hz at each position of interest for a period of 14 seconds. After four presentations at each position, there was a rest period without stimulation for 28 seconds, with each position stimulated 12 times in total. A central fixation point was presented at all times and the participants were instructed to focus on this point.

#### Retinotopic mapping session

Each participant's early visual areas (V1,V2,V3,V4,V3a) were identified in a separate retinotopic mapping session, based on routines developed by Slotnick and Yantis [25].The session consisted of three polar angle mapping runs, and one eccentricity mapping run. Each run consisted of 10 full cycles of rotation (polar angle mapping) or expansion (eccentricity mapping). One cycle was completed in 40 seconds.

#### Main experiments

The stimuli and structure of the two fMRI experiments were identical to the psychophysical experiments. However, to ensure that the hemodynamic response returned to an approximate baseline within areas retinotopically coding a stimulus position between trials, the same position (positions in Experiment 2) was not sampled in adjacent trials. Due to these precautions, the average intertrial interval (the period after the second 2500 ms judgment-period, J2 in Figure 1A and 2A) was only 1 TR (1400 ms), randomly jittered with ±700 ms in two-thirds of the trials [28]. As in the psychophysical experiments, the orientations of the gratings in the S1–S2 task and the F1–F2 task were always orthogonal to each other. Based on the findings from the psychophysical experiments, the two masking conditions were collapsed into one condition: MSR≠1. The mask's spatial frequency was both higher and lower than the sample an equal number of times. The two conditions used in the fMRI experiment, MSR = 1 and MSR≠1, were presented equally often. An experimental run contained 88 trials, and both MSR conditions were sampled 11 times at each stimulus position. Each subject finished 4 runs of each experiment, which were run interleaved and spread over 4 testing sessions, and this produced 176 observations per MSR per experiment. Participants produced their responses using a MR-compatible subject response collection system (ResponseGrip®, NordicNeuroLab, Bergen, Norway).

### MRI data acquisition

Imaging was performed with a Philips Achieva 3 Tesla whole body MR unit equipped with an 8-channel Philips SENSE head coil (Philips Medical Systems, Best, the Netherlands). The functional imaging parameters were the same in the experiments and in the ROI localizer run: 24 transverse slices (no gap) were measured using a BOLD-sensitive T2*-weighted echo-planar imaging (EPI) sequence (repetition time (TR), 1400 ms; echo time (TE), 30 ms; flip angle, 70°; voxel size, 2×2×2 mm; field of view (FOV): 192×192 mm; interleaved acquisition). The imaging parameters for the retinotopic mapping runs were different in some parameters (31 slices; TR, 2000 ms; flip angle, 80°). Anatomical T1-weighted images consisting of 192 sagittally oriented slices were obtained using a turbo field echo pulse sequence (TR, 9.64 ms; TE, 4.59 ms; flip angle 8°; voxel size 1×1×1 mm; FOV, 256×256 mm). A scanning session consisted of 2 experimental runs, and each experimental run produced 890 functional volumes. Before every experimental run, a survey volume with 7 sagittal slices was acquired to place the functional slices along the calcarine fissure of the subject. Between the runs, a transversally oriented version of the whole-brain structural volume with the same coverage as the functional volumes (voxel size 1×1×1 mm; 48 slices) was placed similarly to the functional slices and recorded to facilitate the co-registering of the functional volumes between runs.

### Data analysis

All behavioral data analyses were conducted on a single subject level using paired-samples t-tests to compare accuracy scores from the F1–F2 task over MSR conditions. Due to our strong *a priori* hypotheses about the direction of the effect, one-tailed *p*-values were evaluated. However, it should be noted that visual evidence is considered sufficient in single-subject designs (see for example earlier studies on memory masking; [16], [19]).

Imaging data was pre-processed and analyzed using BrainVoyager QX software (version 2.2., Brain Innovation, Maastricht, The Netherlands). To achieve optimal segmentation results, each participant's individual T1-weighted images (two or more) were corrected for spatial intensity inhomogenities, co-registered, and averaged together to produce a single high-resolution anatomical volume for each participant. These volumes were then transformed into Talairach space, the white-gray matter boundary was estimated and segmented, and bridges were removed using automated procedures in BrainVoyager QX. Based on the white-matter segment of each hemisphere, 3D-meshes of the cortical surfaces were then created. The meshes were inflated, cut along the calcarine fissure, and flattened to get 2D-representations of the cortical surfaces containing each participant's early visual areas. The functional images were first manually inspected - showing sub-millimeter movement for all participants in all runs - then time and motion corrected, co-registered against the individual whole-brain structural volume, and normalized to Talairach space using the transformation parameters estimated from the structural images. Because ROIs were precisely localized for each participant, no spatial smoothing was applied. Univariate statistical analysis based on the General Linear Model – as implemented in BrainVoyager QX – was performed separately for each participant.

Early visual areas were separated based on the polar angle retinotopic maps. Phase encoded maps were computed using a linear cross-correlation analysis and projected on the corresponding flattened cortical surface. The borders of V1, V2, V3, V4, and V3a were then drawn manually, following guidelines provided by Wandell, Dumolin and Brewer [29].

The model representing the ROI localizer task was specified using 4 regressors, each representing the onsets of the flickering stimulus in one of the four positions of interest. The regressors were modeled with durations of 14 seconds and convolved with a two-gamma model of the hemodynamic response function (HRF). Low-frequency drifts were removed using a temporal high-pass filter (cutoff, 0.01 Hz). *t*-contrasts were defined as one regressor against the three others to detect voxels which significantly responded to that position alone. Clusters of voxels larger than 32 mm^3^ that survived a false discovery rate (FDR) correction at *p*<0.01 were separated over early visual areas. Because the ventrally (V4), and dorsally (V3a) confined visual areas both contain a full hemifield representation, each position was represented by five unique ROIs. We also calculated set of ROIs representing the 50 and 100 most spatially selective voxels for each position in each visual area.

The two experimental tasks were represented by two models each. The first model did not separate between error trials (in which a wrong response was given on the F1–F2 task). For Experiment 1 this model was specified using 8 regressors: each regressor started with the onset of the mask stimulus (S1) and lasted until the offset of the second test stimulus (S2), a duration of 9.5 seconds. The regressors were separated over masking conditions (2) and positions (4). For Experiment 2 the first model was created in two versions, representing stimulus presentation to the upper or lower visual field separately (see below). Each model contained 8 regressors: 4 regressors started with the onset of a sample stimulus (F1) and lasted until the offset of the first test stimulus (F2), a duration of 3.5 seconds. These regressors were separated over masking conditions (2) and positions (2). In addition, the model contained 2 regressors modeled as events and representing the onset of the mask stimulus (S1) at each position, and 2 similar regressors representing the onset of the last test stimulus (S2). The second model was similar to the first, except that error trials were represented with a separate set of regressors. Thus, the second set of models representing Experiment 1 and Experiment 2 contained 16 and 12 regressors, respectively. All regressors were convolved with a two-gamma model of the hemodynamic response. The intensity time course at each voxel extracted from the ROIs under investigation were preprocessed following similar routines as Offen et al. [14] and Sligte, Scholte and Lamme [30], also conducting ROI-based univariate analysis on memory-related activity in early visual areas. The time series were temporally smoothed with a high-pass filter of 0.01 Hz and a low-pass filter of 2.8 s, and normalized using z-transformation. For Experiment 1, the resulting average time series from each position ROI within a visual area were combined, producing a single time series consisting of 3560 data points per visual area (890 data points per run × 4 ROIs). For Experiment 2, due to the stimulation of two different positions in a trial (one in the upper and one in the lower visual field), the time series from the visual areas representing the upper and the lower visual field were averaged and analyzed separately. A set of *t*-contrasts were defined *a priori* for each experiment and each model, testing whether the BOLD response to a full trial (Experiment 1), or to stimulus F1 and F2 (Experiment 2), was lower in the memory masking condition (MSR≠1) compared to the MSR = 1 condition. When testing the second model, only correct response trials were included in the contrast. Univariate statistical testing was performed separately on the three sets of localizer-derived ROIs for each visual area. Since the statistical analyses were performed on single time courses, no corrections for multiple comparisons were necessary.

For visualization purposes, and to further investigate the temporal properties of the BOLD-response to the stimuli in a trial, event-related averages were computed for each experimental run, separated over visual areas and the two masking conditions. The underlying time courses were extracted from the set of ROIs derived from the localizer runs when applying the FDR <0.01 threshold. The baseline was calculated as the average of the intensity values at the onset of S1 (Experiment 1), or F1 (Experiment 2), and the two preceding TRs in a run. The calculation of the signal change was performed with this average value following the formula *percent signal change  =  (value - average baseline for run) / average baseline for run*.

## Results

### Behavioral data

The estimated spatial frequency discrimination thresholds at 75% performance level with an ISI of 3 seconds, was a frequency difference of ±11.7% (SD, ± 2.0%) for Participant 1; ±11.3% (SD, ±2.0%) for Participant 2; and ±12.7% (SD, ±3.5%) for Participant 3. At an ISI of 9 seconds, the estimated 85% discrimination threshold was a difference of ±17.7% (SD, ±2.9%) for Participant 1; ±18.7% (SD, ±0.8%) for Participant 2; and ±20.0% (SD, ±1.7%) for Participant 3.

The results from Psychophysical Experiment 1 (Figure 1A), in which all stimuli were presented at the same position in the visual field, are represented in Figure 1B. Both masking conditions produced a memory masking effect on the F1–F2 discrimination task in all participants, significant at the *p*<0.05 level (*t*(2)>2.92, one-tailed *p*-value). The behavioral results from Experiment 1 conducted in the MR-scanner replicated the psychophysical results (Figure 1C). The hit rates in the masking condition MSR≠1 (MSR = 2 and MSR = 0.5 collapsed) were significantly lower in all participants than the hit rates in the MSR = 1 condition (*t*(3)>2.35, *p*<0.05, one-tailed *p*-value).

The results from Psychophysical Experiment 2 (Figure 2A), in which the masking stimulus S1 and the sample stimulus F1 were presented in opposite visual quadrants, are presented in Figure 2B. No significant memory masking effect was observed for two of the participants (Participant 1 and 3) in Psychophysical Experiment 2, however Participant 2 showed a strong trend (*p*<0.1) towards a masking effect in the MSR = 2 condition. One participant did show a significantly lower accuracy in the MSR = 2 condition (Participant 2, *t*(3)>2.35, *p*<0.05, one-tailed *p*-value), and a strong trend (*p*<0.1) towards a masking effect in the MSR = 0.5 condition. The behavioral results from the Experiment 2 conducted in the MR-scanner were similar: Participant 1 and 3 did not show any significant masking effects, while Participant 2 showed a significantly lower accuracy in the MSR≠1 condition (Figure 2C).

### fMRI data

**Figure 3:**
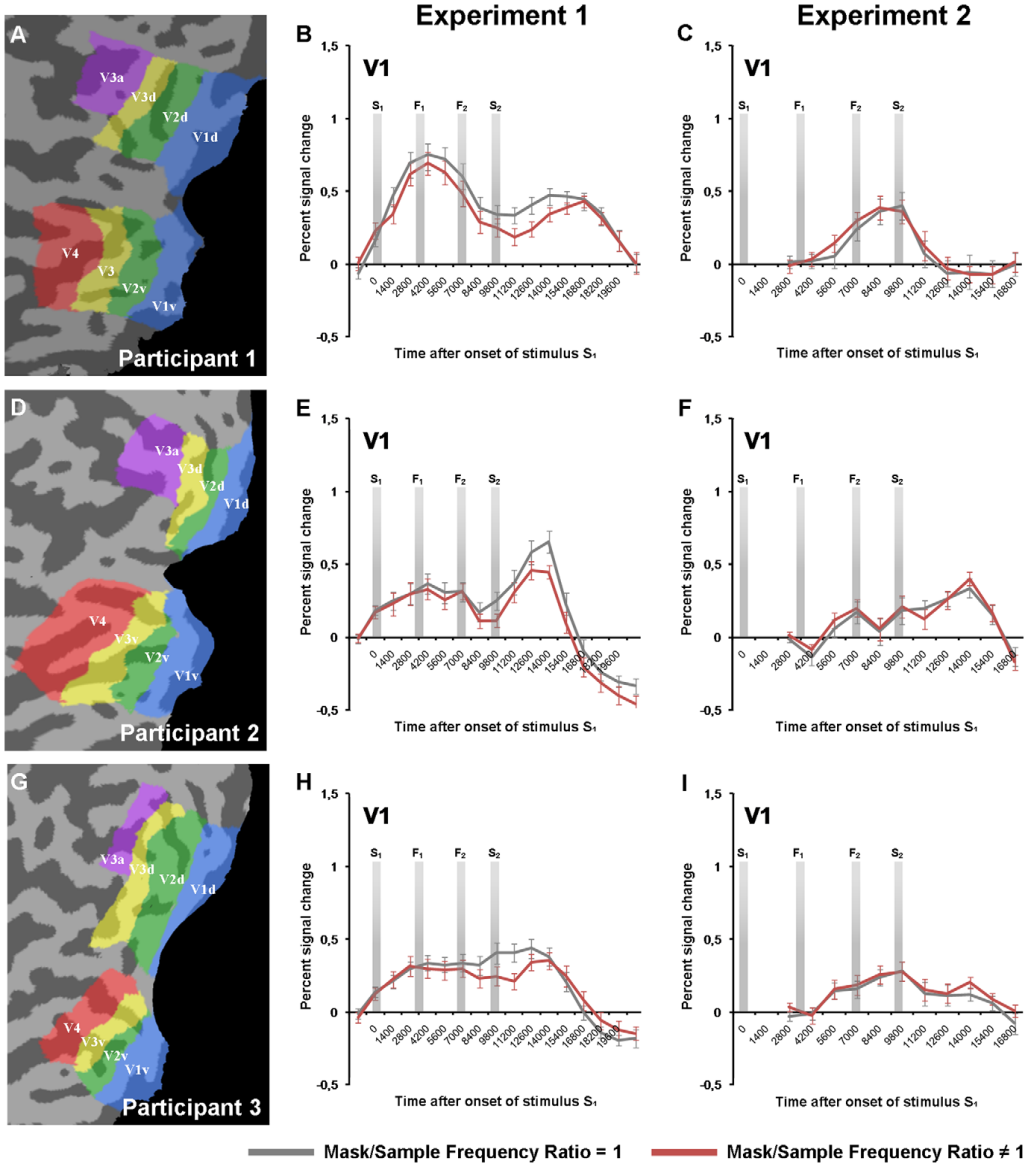
Event-related averages, V1. A, D, G) Early visual areas investigated in the fMRI analysis. The figures show flattened representations of each participant's left visual cortex. Regions of interest (ROIs), representing voxels sensitive for the different stimulus locations used in the experiments, were defined separately across visual areas based on data from a separate localizer scan. B, E, H) Event-related averages from participant 1–3′s V1 ROIs in Experiment 1. The data shown here were extracted from V1 ROIs defined as voxels from the localizer data surviving a FDR <0.01 threshold (see the methods). All trials were included in the creation of the time series (corresponding to our statistical model 1).The vertical bars in each plot represent the stimulus onsets in a trial. C, F, I) Event-related averages from Experiment 2, extracted from the same V1 ROIs as for Experiment 1. Since stimulus S_1_ and S_2_ were presented to the opposite position of stimulus F_1_ and F_2_, the curves represent the activity from the V1 ROIs coding for the F_1_/F_2_-position. Error bars represent ±1 SE.

**Figure 4:**
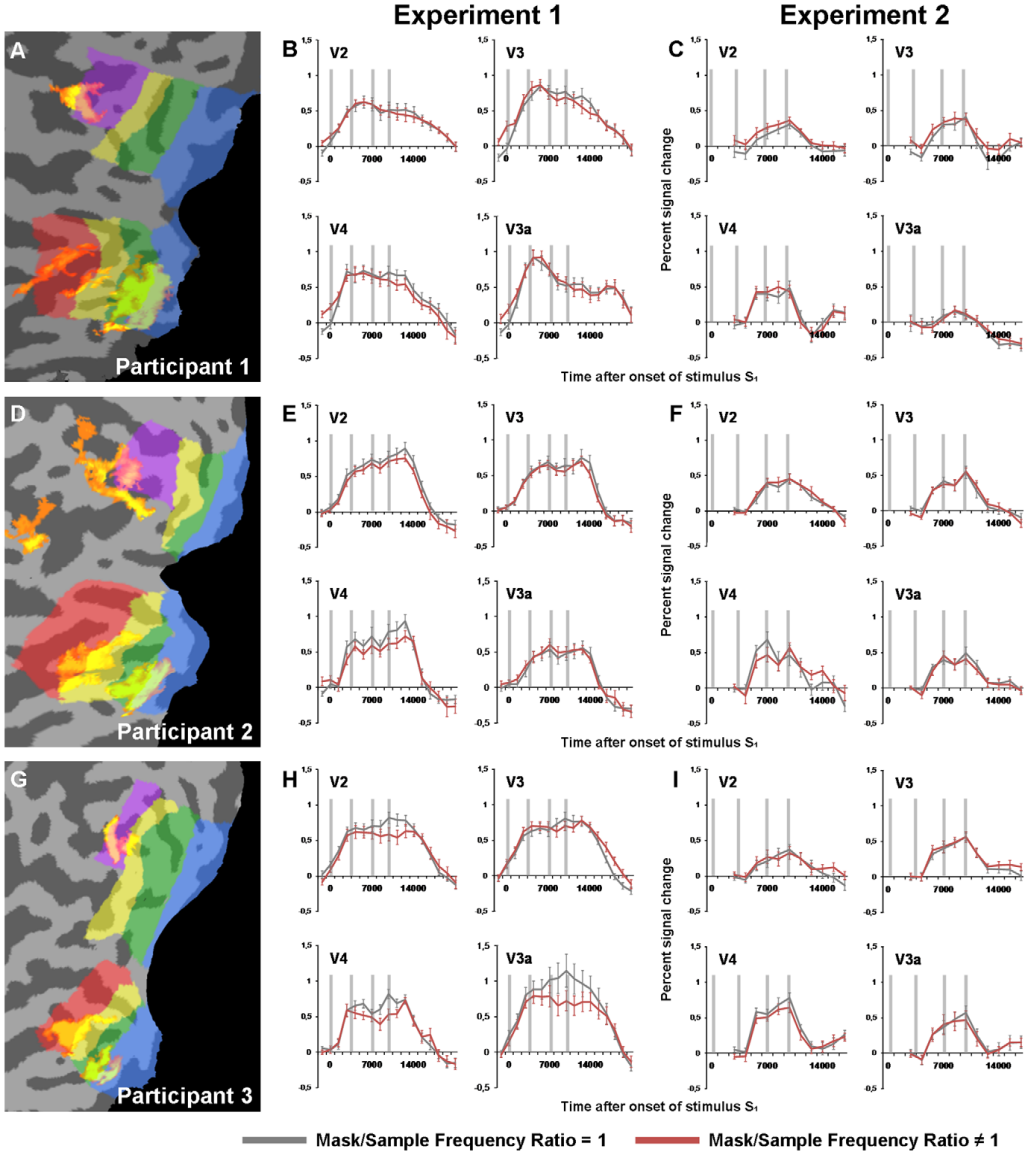
Event-related averages, V2-V3a. A, D, G) Examples of activation maps from the ROI localizer scan. The activity clusters represent voxels sensitive to the stimulus position in the upper right visual field (thresholded at FDR <0.01). The resulting ROIs were defined as active voxels overlapping with the different visual areas. B, E, H) Event-related averages from the participants’ V2, V3, V4 and V3a ROIs in Experiment 1. The data shown here were extracted from ROIs defined as voxels from the localizer data surviving a FDR <0.01 threshold (similar to Figure 3). C, F, I) Event-related averages from Experiment 2, extracted from the same V2-V3a ROIs as for Experiment 1. Since stimulus S_1_ and S_2_ were presented to the opposite position of stimulus F_1_ and F_2_, the curves only represent the activity from ROIs coding for the F_1_/F_2_-positions.

All fMRI data analyses were conducted on intensity time courses extracted from each participant's individual ROIs in early visual areas. The sizes of these ROIs are presented in Table 1. We defined the early visual areas V1, V2, V3, V4 and V3a based a separate retinotopic mapping session. The resulting individual visual areas are depicted on flattened versions of the occipital cortex in Figure 3 (A,D,G), while activation maps from the ROI-localizer are shown in Figure 4 (A,D,G).

**Table 1:** Number of voxels constituting individual ROIs (FDR <0.01 threshold).

	Average ROI size in 32 mm^3^ voxels ± sd				
	V1	V2	V3	V4	V3a
Participant 1	92±21	104±19	44±24	27±14	41±9
Participant 2	117±53	88±41	57±32	16±10	36±19
Participant 3	82±29	125±85	88±41	47±41	34±19

sd – standard deviation

For Experiment 1, the contrast of interest was between the measured BOLD amplitudes for the full train of stimuli in a trial in the two main conditions: MSR = 1 and MSR≠1. In V1, with localizer-derived V1 ROIs thresholded at FDR<0.01, all participants showed a significantly lower response in the MSR≠1 condition when all trials were included in the analysis (Model 1: Participant 1, *t* = 3.0, *p*<.01; Participant 2, *t* = 2.2, *p*<.05; Participant 3, *t* = 5.3, *p*<.01). Event-related averages representing the time series of the two conditions are depicted in Figure 3 (B,E,H). Similar results were also found when only trials in which the participants successfully discriminated F2 from F1 were included in the analysis (Model 2: Participant 1, *t* = 2.7, *p*<.01; Participant 2, *t* = 2.6, *p*<.01; Participant 3, *t* = 4.7, *p*<.01). Finally, the analyses of both models produced the same significant results in all participants when the V1 ROIs analyzed consisted of the 50 or 100 most spatially selective voxels for each position. The other visual areas (V2–V3a) did not show a similar consistent differential pattern of activity across participants. No significant differences were found in Participant 1 for any of the models across the different ROI definitions. Participant 2 showed significant masking effects in V2 for both models when the V2 ROIs were defined as the 50 most spatially selective voxels (Model 1 & Model 2: *t*>2.8, *p*<.01). Participant 3 showed significant masking effects in V4 (Model 1 & Model 2: *t*>3.0, *p*<.01) and V3a (Model 1 & Model 2: *t*>4.0, *p*<.01) for the FDR <0.01 ROI definition, and similar effects was observed when analyzing the 50 or 100 most spatially selective voxels for each position. Importantly, no significant effects were found in the analyses of Model 2, in which only correct response trials were included, that were not found in the analyses of Model1. Event-related averages representing the time series of the two conditions in visual areas V2–V3a are depicted in Figure 4 (B,E,H).

For Experiment 2, the contrast of interest was between the measured BOLD amplitudes to stimulus F1 and F2 in the two main conditions: MSR = 1 and MSR≠1. Due to stimulation across the horizontal meridian in a trial in Experiment 2, the ROIs coding for the upper and lower visual field were analyzed separately (see the *Methods* part). In V1, none of the participants showed any significant differences between the conditions in any of the models, nor with any of the different criteria for defining the V1 ROI. Event-related averages representing the V1 BOLD time series after the onset of stimulus F1 are depicted in Figure 3 (C,F,I). As with Experiment 1, no consistent differential pattern of activity across participants was found in the analysis of the visual areas V2–V3a. Event-related averages representing the time series in visual areas V2–V3a after the onset of stimulus F1 are depicted in Figure 4 (C,F,I).

## Discussion

We observed memory masking effects consistent with earlier studies [16], [17], [18], [19], [31] in all participants when the mask was presented to the same position in the visual field as the stimuli constituting the main discrimination task (Psychophysical and fMRI Experiment 1). Memory masks that differed in spatial frequency from the sample stimulus with ±1 octave impaired the participant's discrimination performance. This masking effect was reflected in all participants as a lower BOLD response in V1 voxels coding for the stimulus position in a trial. As can be seen in Figure 3 (panels B,E,H), this effect starts (i.e. the time series separate reliably) 5–6 seconds after the presentation of the sample stimulus (F1), an observation that fits well with the temporal delay inherent in the BOLD response [32]. Note that the two conditions only varied in the spatial frequency ratio between the mask and the sample stimulus (MSR = 1, MSR = 0.5, or MSR = 2), and that the participants were unaware of this relationship until the presentation of the sample stimulus. Thus any condition-dependent effect could not appear before this time point. Following recent models of sensory visual working memory [4], [6], [20], we interpret this finding as a result of cross-channel interactions between neural populations coding for different ranges along the spatial frequency spectrum. Since the interacting representations are separated in time, we further take this finding as evidence for the recruitment of V1, the earliest stage in cortical processing of visual input, in memory for spatial frequencies. This interpretation is in agreement with findings from recent fMRI studies on memory for low-level visual features: using multivariate analysis approaches, Harrison and Tong [7], and Serences et al. [8] managed to decode featural attributes of sample stimuli (orientation/orientation + color, respectively) from patterns of activity in V1 during delay periods. The conclusions one can draw from MVPA are however limited by the low differential resolution of the classification procedures; the classifier algorithms can only distinguish between categories they have been explicitly trained on. Low-level memory representations, on the other hand, are stored with impressive precision [5], and the representations that participants can discriminate between in studies on low-level memory could not have been distinguished using MVPA. Consequentially, the decoding in the two above-mentioned studies was performed on trial differences that were easy to categorize (e.g. orientations differing with 90° [7]), while the behavioral discrimination task within a trial typically was performed on differences set at 75% discrimination threshold (±3–6° [7]). Whether the observed delay activity represents the task-relevant, high fidelity, memory trace or is due to (or the same as) other sensory-recruitment processes such as feature-based attention [33], [34] or visual imagery [35], [36], [37], is therefore difficult to decide using multivariate approaches. Here we argue that an univariate analysis approach, which allows intensity comparisons on a continuous measurement scale, is an important supplement to the categorical differentiation used in MVPA when studying the brain's processing of fine-grained differences between representations.

Harrison and Tong [7], and Serences et al [8], also analyzed the data using standard univariate, intensity-based analyses without finding any evidence of sustained V1 activity during the delay period. One possible reason for this, suggested by Offen et al. [14] after a similar finding, is that the activation of neurons sensitive for the specific value of the remembered feature leads to a suppression of neurons tuned to other values, leaving the population average unchanged. Our finding shows that such processes take place, but while the cross-channel interactions cancel out the memory-related neural activity on the measured voxel level in standard delayed discrimination tasks, these interactions manifest as a relative weakening of the BOLD signal in masked trials in our study. In effect, the two conditions in our memory masking paradigm produce an amplitude contrast which is measurable using univariate analysis approaches – resulting from the sample stimulus activating neurons from a suppressed population in one condition (MSR≠1), and neurons from a population unaffected by the mask stimulus in the other condition (MSR = 1).

The results from the analysis of Experiment 1 in the other visual areas (V2-V3a) were not consistent across participants, thus all reported findings from these areas were done at an N = 1 level, making it difficult to interpret their significance with respect to the memory masking effect. Differences concerning the involvement of extrastriate areas in low-level memory is also evident from the results in the recent studies discussed: some researchers, applying a decoding approach [8], [21], or univariate analyses [14] did not find any memory-related effects in these areas, while others [7] managed to decode feature-specific activity patterns in all visual areas investigated (combining area V4 and V3a). Differences in stimulus salience and other task-specifics, as well as factors more difficult to control such as task-solving strategies applied by the individual participants, might explain this discrepancy across and within studies. Furthermore, the peripherally presented stimuli used in our experiment (see also [21]), produced relatively small clusters of significant position-selective voxels in some extrastriate areas (in particular V4 and V3a), perhaps leading to a higher susceptibility to noise in these areas compared to the larger V1-ROIs.

In addition to analyzing data extracted from ROIs defined based on a statistical threshold (FDR <0.01, see the methods), we investigated data from the 50 and 100 most spatially selective voxels for each stimulus position. This implied a stricter criterion for some visual areas, and a less stringent threshold for others; nevertheless, V1 was still the only area that showed consistent masking effects across participants. Still, when investigating the event-related averages from area V2–V3a (Figure 4, panels B, E, H), there seems to be some indications of an effect of masking in V4 in all participants. This observation is consistent with a finding from Bennet and Cortese [38], showing that memory masking is selective to the perceived rather than the retinal spatial frequency when the stimuli are presented at different distances, suggesting that the mechanisms behind memory masking includes processing levels involved in the computation of size and shape constancies (e.g. V4 [39], [40]). These processes have however been found to modulate V1 activity as well, possibly through cortical feedback processes [41], [42].

The observed memory modulation of V1 was consistent over the two models tested in all participants. Only correct trials were included in the comparison of V1 BOLD data over different mask/sample frequency ratio condition in the second model, thus, error-related processes, which were more common for masked stimuli, cannot explain the activity differences in Experiment 1. Due to the manipulation of orientation between mask and sample stimuli, the observed effect is also not likely to be caused by repetition priming - observed as higher BOLD responses in V1 for the second presentation of a stimulus [43], [44]. At short intervals, priming effects can be replaced with the effect of neural adaptation, which is a decrease in neural sensitivity over repeated stimulations [45]. However, stimuli with short durations (<1 sec) do not affect V1 responses to later stimuli [46], and effects are only evident at very short ISIs [47]. In any case, adaptation leads to reduced neural responses when two successive stimuli activate the same subpopulation; as a result, any effects from neural adaptation in our study would produce the opposite pattern of activation between conditions compared to what was observed. Attentional effects are also known to affect the BOLD response in early visual areas (e.g. [48]). Participants may be able to detect when the mask and sample share the same spatial frequency (MSR = 1), and thereby devote more attentional resources to the processing of the sample stimulus. We do however find this explanation unlikely: although discrimination thresholds increase, it is possible to discriminate (and therefore recognize similarities between) spatial frequencies of stimuli with different positions in the visual field with high precision [49]. Nevertheless, we only find V1 modulations when the mask and sample stimuli are presented to the same position (Experiment 1).

Our second experiment (Psychophysical and fMRI Experiment 2) investigated the spatial extent of the memory masking effect and the underlying spatial frequency memory representations. We found weakened or absent behavioral memory masking effects in all participants when the mask and sample stimuli were presented to opposite parts of the visual field; however, strong trends toward significant memory masking were still present in the behavioral data. Due to these residual effects, we cannot conclude that memory masking is a strictly retinotopically confined process. Since the fMRI setup in our study was optimized for studying the early visual cortex, our data does not let us investigate the contribution to memory masking from other parts of the brain known to play a role in the storage of position information in visual working memory (see for example [50]). However, as discussed above, the memory masking phenomenon is likely to involve processing levels responsible for the calculation of size and shape constancies [38], thus the observed effects in Experiment 2 might be due to competition between representations in non-retinotopically organized parts of the brain.

Nevertheless, the fMRI results from Experiment 2 in the early visual areas shows some interesting patterns with respect to the memory masking phenomenon: No differences were observed in the measured BOLD response in V1. The absence of a difference between conditions in V1 (Figure 3, panels C, F, I) compared to Experiment 1, suggests that the memory masking effect at least partially is caused by interactions between representations with limited spatial extent. Interestingly, a recent investigation of V1 population responses to superimposed gratings of different orientations, well-known to produce an increase in perceptual detection thresholds (cross-orientation suppression; see [51] for a short review), show that this population activity can be modeled as the average of the responses to the component gratings [52] (see also [53] for similar findings from visual evoked potentials in human V1). Thus, neurons preferring one of the orientations are suppressed by neurons preferring the other orientation, and vice versa. The researchers further show that this effect does not occur when the stimuli are separated by more than the neurons' receptive fields, a finding in line with the observation of a V1 memory masking effect in Experiment 1, but not in Experiment 2. This observation also touches upon a related issue concerning the spatial specificity of the V1 memory representations: Ong et al. [23] conducted an experiment on the effects of spatially separating sample and test stimuli in a delayed discrimination task for direction of motion. They discovered that the discrimination thresholds increased when the stimuli were presented to different positions, but only if the distance was larger than the receptive field size of V5/MT-neurons coding for the given eccentricity (see also [22], Experiment 2). Thus, comparison at a distance can be performed, but it seems to require a transfer of information from one set of neurons to another, a process introducing noise and affecting performance. The same conclusion can be drawn regarding features coded in V1: Danilova and Mollon [49] report delayed discrimination thresholds for spatially separated spatial frequency and orientation stimuli that are markedly higher than thresholds reported when sample and test are presented to the same position [18]. A recent fMRI study, however, applying multivariate analysis methods to investigate the spatial extent of sensory recruitment in a delayed discrimination task for orientation, found a global spread of activation during the retention phase representing the approximate angle of the remembered stimulus [21]. Specifically, the researchers decoded this information from ipsilateral V1 relative to stimulus position, i.e. in the opposite hemisphere of the neurons retinotopically coding for the remembered item. As discussed above, other sensory recruitment processes have been shown to produce similar patterns of activity: feature-based attention, for example, can produce global modulations of measured V1 activity, even in the absence of visual stimulation [34], [54]. We therefore speculate that different processes are involved at the global and retinotopic level during retention of low-level attributes: at the global level neurons tuned to the attended dimensions are modulated to increase sensitivity for the task-relevant aspect of the stimuli, while the high fidelity memory trace in V1 is confined retinotopically.

One might question our approach of analyzing the conditions in our experiment as blocks, as it makes us unable to investigate the different processes constituting a trial separately. Since the stimuli in our experimental paradigm have to follow in fixed order, however, the necessary design precautions to allow disentangling of the different contributions would either involve very long inter-stimulus intervals, or an enormous number of partial trials [55], [56], [57], in either case, making the paradigm too long to be compatible with a fMRI approach. Anyhow, our main finding; modulation of V1 activity during memory for the visual low-level feature spatial frequency, complements recent findings from multivariate approaches, and further suggests that memory representations result from the recruitment of the same neural populations that were involved in the sensory encoding of the remembered stimulus.
